# Supplementary material for: Age-related disparities in clinical characteristics and outcomes of patients with severe fever with thrombocytopenia syndrome
Source: PLoS Negl Trop Dis. 2025 Nov 4;19(11):e0013694. doi: 10.1371/journal.pntd.0013694 (PMC12604809; doi:10.1371/journal.pntd.0013694)
Supplement: S1 Checklist — The filled checklist is based on the STROBE Statement-Checklist of items that should be included in reports of observational studies, developed by the STROBE Initiative, https://www.strobe-statement.org/ (DOC) [file pntd.0013694.s001.doc]

**S1 Checklist. STROBE checklist.**

|  | Item No | Recommendation |
| --- | --- | --- |
| **Title and abstract** | 1 | (*a*) Age-related disparities in clinical characteristics and outcomes of patients with severe fever with thrombocytopenia syndrome |
|  |  | (*b*) Background and Aim: The exploration of age-related clinical characteristics and prognosis of severe fever with thrombocytopenia syndrome (SFTS) has not been largely addressed in current research. This study aimed to analyze the differences in clinical features and outcomes of SFTS patients across various age groups.  Methods: Data on demographics, comorbidities, clinical manifestations, laboratory test results, complications, treatment, and outcomes of patients with SFTS were retrospectively collected. Patients were assigned to four groups: age ≤ 54 years, age 55–64 years, age 65–74 years, and age ≥ 75 years, and then their clinical data were compared.  Results: A total of 253 patients diagnosed with SFTS were retrospectively included. Compared with patients aged <65 years, patients aged ≥65 years had a higher proportion of male, more presence of lethargy, chest distress, dyspnea, gingival bleeding, and myasthenia. Patients aged ≥65 years had higher serum levels of laboratory parameters referring to liver, kidney, heart, and coagulation system injury, as well as higher viral load. Moreover, the serum levels of procalcitonin, SAA, ferritin, IL-6, IL-10, and TNF-α were significantly higher, but the CD3+ and CD3+ CD4+ lymphocytes percentage and counts were significantly lower in patients aged ≥65 years than in patients aged <65 years. The cumulative survival rate of patients aged ≥65 years was also significantly lower than that of patients aged <65 years. The univariate and multivariate logistic regression analyses identified that age ≥65 years was an independent risk factor for the prognosis of patients with SFTS.  Conclusion: Age significantly influences the clinical features and prognosis of patients, and age ≥65 years is associated with adverse outcomes in patients with SFTS. Personalized management might be required among different age groups. |
| Introduction | | |
| Background/rationale | 2 | The exploration of age-related clinical characteristics and prognosis of severe fever with thrombocytopenia syndrome (SFTS) has not been largely addressed in current research. |
| Objectives | 3 | This study aimed to analyze the differences in clinical features and outcomes of SFTS patients across various age groups. |
| Methods | | |
| Study design | 4 | A retrospective observational study |
| Setting | 4 | According to the age distribution of patients, they were assigned to four groups with a 10-year age interval, centered at 55, 65, and 75 years. The age ranges for each group were as follows: Group1 (G1) <55 years, n=36; Group2 (G2) 55–64 years, n=75; Group3 (G3) 65–74 years, n=105; Group4 (G4) ≥ 75 years, n=37 |
| Participants | 4 | A total of 253 patients with SFTS admitted to the Department of Infectious Diseases, Zhongnan Hospital of Wuhan University between August 2016 and October 2024 were included in a retrospective cohort. |
| Variables | 5 | The inclusion criteria were as follows: (1) patients with fever (temperatures ≥ 37.3 ˚C); (2) patients with decreased platelet count; (3) patients with positive results of serum SFTSV RNA via reverse transcriptase polymerase chain reaction. Patients were excluded if they fulfilled one or more of the following reasons: (1) the presence of preterminal comorbidities (heart disease New York Heart Association III–IV, severe chronic obstructive pulmonary disease, chronic renal failure), (2) any other types of immunodeficiency, (3) history of malignant tumor, (4) incomplete clinical data or missed follow-up. (5) the presence of other viral infections such as coronavirus disease 2019 (COVID-19). |
| Data sources/ measurement | 6* | The medical records of patients with SFTS were reviewed, demographic details, comorbid conditions, symptoms, and laboratory test results including white blood cell (WBC) count and percentage, neutrophil count and percentage, lymphocyte count and percentage, platelet count, hemoglobin, alanine aminotransferase (ALT), aspartate aminotransferase (AST), total bilirubin (TBIL), albumin, alkaline phosphatase (ALP), gamma glutamyl transpeptidase (GGT), lactate dehydrogenase (LDH), total cholesterol (TC), triglyceride (TG), blood urea nitrogen (BUN), creatinine, cystatin-C, sodium, potassium, amylase, lipase, creatinine kinase (CK), creatinine kinase myocardial b fraction (CK-MB), troponin I, brain natriuretic peptide (BNP), prothrombin time (PT), international normalized ratio (INR), prothrombin activity (PTA), activated partial thromboplastin time (APTT), thrombin time (TT), fibrinogen, D-dimer, SFTSV viral load, occult blood test (OBT) , C-reactive protein (CRP), procalcitonin, erythrocyte sedimentation rate (ESR), serum amyloid A (SAA), ferritin, interleukin-2 (IL-2), interleukin-4 (IL-4), interleukin-6 (IL-6), interleukin-8 (IL-8), interleukin-10 (IL-10), tumor necrosis factor-α (TNF-α), interferon-γ (IFN-γ), CD3+ lymphocytes count and percentage, CD3+ CD4+ lymphocytes count and percentage, CD3+ CD8+ lymphocytes count and percentage, CD19+ lymphocytes count and percentage, CD16+CD56+ cells count and percentage were collected. Some severe complications, including respiratory failure, acute kidney injury, acute pancreatitis, shock, systemic inflammatory response syndrome (SIRS), and bacterial or fungal infections, disseminated intravascular coagulation (DIC), rhabdomyolysis, and haemophagocytic lymphohistiocytosis (HLH), were monitored and recorded during hospitalization. The treatment prescriptions of hospitalized patients, including noninvasive mechanical ventilation, invasive mechanical ventilation, continuous renal replacement therapy, use of vasoactive drugs, use of corticosteroids, intravenous immunoglobulin treatment, and platelet transfusion, were reviewed and recorded in the cohort. The primary outcome was in-hospital mortality. The follow-up time of survivors was calculated from the day of admission to recovery, and the survival time of non-survivors was calculated from the day of admission to that of death. |
| Bias | 5 | This was a retrospective single-center study, the observations made here could not be extrapolated to other centers. |
| Study size | 4 | A total of 253 patients with SFTS admitted to the Department of Infectious Diseases, Zhongnan Hospital of Wuhan University between August 2016 and October 2024 were included in a retrospective cohort. |
| Quantitative variables | 6 | The medical records of patients with SFTS were reviewed, demographic details, comorbid conditions, symptoms, signs, radiological findings and laboratory tests data were collected. |
| Statistical methods | 7 | The quantitative data were shown as medians with interquartile ranges (P25-P75) for data with a non-normal distribution and compared by the Kruskal-Wallis method. The qualitative data were shown as numbers (percentages) and evaluated by the Chi-square test or Fisher’s exact test. The cumulative survival rates, SFTSV RNA positive rates, and platelet count normalization rates of patients across four age groups were evaluated using the Kaplan-Meier method and compared by the Log-rank test. Univariate and multivariate logistic regression analyses were performed to identify independent factors for in-hospital mortality of patients with SFTS. All data were analyzed with SPSS statistical analysis software version 26.0 (IBM), and P < 0.05 (two-sided) was considered statistically significant. |
| Results | | |
| Participants | 4* | According to the age distribution of patients, they were assigned to four groups with a 10-year age interval, centered at 55, 65, and 75 years. The age ranges for each group were as follows: Group1 (G1) <55 years, n=36; Group2 (G2) 55–64 years, n=75; Group3 (G3) 65–74 years, n=105; Group4 (G4) ≥ 75 years, n=37 |
| Descriptive data | 6* | The treatment prescriptions of hospitalized patients, including noninvasive mechanical ventilation, invasive mechanical ventilation, continuous renal replacement therapy, use of vasoactive drugs, use of corticosteroids, intravenous immunoglobulin treatment, and platelet transfusion, were reviewed and recorded in the cohort. |
| Outcome data | 6* | The primary outcome was in-hospital mortality. The follow-up time of survivors was calculated from the day of admission to recovery, and the survival time of non-survivors was calculated from the day of admission to that of death. |
| Main results | 7-12 | The median age of included patients was 66 years (interquartile range [IQR], 59–72 years), and the age distribution of patients in the study was shown in Fig 1. The median age of the four groups (G1-G4) was 52 years (IQR, 51–53 years) for G1, 60 years (IQR, 57-63 years) for G2, 70 years (IQR, 68-72 years) for G3, and 79 years (IQR, 77-80 years) for G4, respectively. Demographic characteristics, comorbidities, and clinical symptoms were compared among the four age groups of patients with SFTS. The findings revealed that patients in the G3 and G4 had a higher proportion of male and more presence of lethargy, chest distress, dyspnea, gingival bleeding, and myasthenia than patients in the G1 and G2. However, there was no significant difference in the prevalence of diabetes and hypertension, frequencies of coma, confusion, dysphoria, cough, sputum, gastrointestinal symptoms, and other haemorrhagic events except for gingival bleeding, fever >38 ℃, chills, headache, myalgia, and dizziness among the four groups. Among these laboratory parameters, serum levels of AST, LDH, BUN, creatinine, cystatin-C, potassium, CK, CK-MB, troponin I, and BNP of patients in the G3 and G4 were significantly higher than those of patients in the G1 and G2. In contrast, serum levels of albumin of patients in the G3 and G4 were lower than those of patients in the G1 and G2. No significant differences were observed among the four groups for the remaining laboratory variables. The serum levels of acute-phase proteins, inflammatory cytokines, and immune cells counts and percentages were also compared among the four age groups of patients with SFTS. As shown in Table 3, patients in the G3 and G4 had significantly higher serum levels of procalcitonin, SAA, ferritin, IL-6, IL-10, and TNF-α than patients in the G1 and G2. Additionally, the CD3+ lymphocytes, especially CD3+ CD4+ lymphocytes counts and percentages in the G3 and G4 were significantly lower than those in the G1 and G2. In terms of complications, patients in the G3 and G4 had more presence of respiratory failure, acute kidney injury, shock, SIRS, bacterial or fungal infections, and DIC than those in the G1 and G2. There were no statistical differences in the incidence of acute pancreatitis, HLH, and rhabdomyolysis among the four groups. During hospitalization, patients in the G3 and G4 were treated with a higher proportion of noninvasive mechanical ventilation, invasive mechanical ventilation, continuous renal replacement therapy, use of vasoactive drugs, and platelet transfusion compared to those in the G1 and G2. No significant differences were observed in the use of corticosteroids and intravenous immunoglobulin treatment among the four groups |
| Other analyses | 12-14 | The SFTSV RNA positive rates and platelet count normalization rates of SFTS patients across four age groups were compared after hospitalization, respectively.  The median duration of SFTSV RNA shedding was 10 days (IQR, 8–15 days), 12 days (IQR, 8–16 days), 15 days (IQR, 10–18 days), and 16 days (IQR, 12–20 days) of patients in the G1, G2, G3, and G4, respectively. The results showed that the SFTSV RNA positive rates of patients in the G3 and G4 were significantly higher than those in the G1 and G2 after hospitalization (Fig 2A). The median duration of platelet count recovering to normalization was 16 days (IQR, 12–19 days), 17 days (IQR, 12–22 days), 19 days (IQR, 15–24 days), and 21 days (IQR, 17–26 days) of patients in the G1, G2, G3, and G4, respectively. The findings indicated that the platelet count normalization rates of patients in the G3 and G4 were significantly lower than those in the G1 and G2 after hospitalization (Fig 2B). The cumulative survival rates of patients in the G1, G2, G3, and G4 were 94.4%, 89.3%, 74.3%, and 73.0%, respectively (Fig 3). The cumulative survival rates of patients in the G3 and G4 were significantly lower than those in the G1 and G2 (P = 0.006). Demographics, clinical manifestations, comorbidities, and laboratory parameters were included in the univariate and multivariate logistic regression analyses to explore the independent risk factors for in-hospital mortality of SFTS patients. In the univariate analysis, age ≥65 years, neurological manifestations, dyspnea, abdominal pain, myalgia, hemoglobin, platelet count, AST, albumin, LDH, TC, BUN, creatinine, cystatin-C, potassium, amylase, lipase, CK-MB, myoglobin, BNP, PT, PTA, APTT, TT, fibrinogen, D-dimer, viral load, and OBT positivity were associated with mortality. Further multivariate analysis could be summarized as follows: age ≥65 years (adjusted odds ratio (aOR), 4.019; 95% confidence interval (CI): 1.568–10.304, P = 0.004), neurological manifestations (aOR, 3.408; 95% CI: 1.415–8.211, P = 0.006), creatinine (aOR, 1.008; 95% CI: 1.004–1.012, P <0.001), PT (aOR, 1.388; 95%CI: 1.024–1.882, P = 0.034), and APTT (aOR = 1.055; 95%CI: 1.023–1.089, P = 0.001), which could serve as independent risk factors for in-hospital mortality of SFTS patients. |
| **Discussion** |  |  |
| Key results | 16 | This study aimed to compare the clinical characteristics of SFTS patients across different age groups and to explore the impact of age on the adverse outcomes in these patients. The results demonstrated significant variations in gender, clinical manifestations, and laboratory parameters of patients with SFTS across different age groups. Most importantly, we revealed that both serious complications and poor prognosis risks also showed significant increases with age, especially in patients aged ≥65 years. It was also shown that age ≥65 years was an independent risk factor for in-hospital mortality of patients with SFTS. These findings provide a theoretical framework for considering the effects of age on the formulation of primary treatment strategies for SFTS. |
| Limitations | 19 | The main limitations of our study include the retrospective study design. Additionally, the sample size of this study was relatively small, leading to limited generalizability of the findings. |
| Interpretation | 19 | In summary, our study revealed significant disparities in gender, clinical manifestations, laboratory test results, complications, and treatment among SFTS patients across different age groups. Notably, patients aged 65 and above were more prone to multiple organ dysfunction and serious complications, resulting in a high in-hospital mortality. |
| Generalisability | 19 | Clinicians should attach importance to these age-specific features and conduct individualized intervention strategies to improve the prognosis of patients with SFTS. Additionally, further study can explore the differences in risk factors for poor prognosis of SFTS patients across different age groups, providing a theoretical basis for effective therapeutic strategies tailored to different age populations. |
| **Other information** |  |  |
| Funding | 20 | This work was supported by the Discipline Cultivation Funding, Zhongnan  Hospital of Wuhan University (XKJS202025). The funders had no role in the study design, data collection and analysis, decision to publish, or preparation of the manuscript. |

*Give information separately for cases and controls in case-control studies and, if applicable, for exposed and unexposed groups in cohort and cross-sectional studies.

**Note:** An Explanation and Elaboration article discusses each checklist item and gives methodological background and published examples of transparent reporting. The STROBE checklist is best used in conjunction with this article (freely available on the Web sites of PLoS Medicine at http://www.plosmedicine.org/, Annals of Internal Medicine at http://www.annals.org/, and Epidemiology at http://www.epidem.com/). Information on the STROBE Initiative is available at www.strobe-statement.org.
