# Supplementary material for: Age-related disparities in clinical characteristics and outcomes of patients with severe fever with thrombocytopenia syndrome
Source: PLoS Negl Trop Dis. 2025 Nov 4;19(11):e0013694. doi: 10.1371/journal.pntd.0013694 (PMC12604809; doi:10.1371/journal.pntd.0013694)
Supplement: S1 Table — (DOCX) [file pntd.0013694.s002.docx]

**S1 Table. The normal range of laboratory parameters.**

|  | Normal range |  |
| --- | --- | --- |
|  |  |  |
| WBC (10^9^/L) | 3.5-9.5 |  |
| Neutrophil (%) | 40.0-75.0 |  |
| Neutrophil (10^9^ /L) | 1.8-6.3 |  |
| Lymphocyte (%) | 20.0-50.0 |  |
| Lymphocyte (10^9^/L) | 1.1-3.2 |  |
| Hemoglobin (g/L) | 130-175 |  |
| Platelet (10^9^ /L) | 125-350 |  |
| ALT (U/L) | 9-50 |  |
| AST (U/L) | 15-40 |  |
| TBIL(μmol/L) | 5-21 |  |
| Albumin (g/L) | 40-55 |  |
| ALP (U/L) | 30-120 |  |
| GGT (U/L) | 8-57 |  |
| LDH (U/L) | 125-243 |  |
| TC (mmol/L) | 0-5.18 |  |
| TG (mmol/L) | 0-1.70 |  |
| BUN (mmol/L) | 2.8–7.6 |  |
| Creatinine (mmol/L) | 64-104 |  |
| Cystatin-C (mg/L) | 0-1.2 |  |
| Potassium (mmol/L) | 3.5-5.3 |  |
| Sodium (mmol/L) | 137-147 |  |
| Amylase (U/L) | 0-90 |  |
| Lipase (U/L) | 0-70 |  |
| CK (U/L) | 0-171 |  |
| CK-MB (U/L) | 0-25 |  |
| Myoglobin(ng/ml) | 0-140.1 |  |
| Troponin I (pg/mL) | 0-26.2 |  |
| BNP (pg/mL) | 0-100 |  |
| PT (s) | 9.4-12.5 |  |
| INR | 0.85-1.15 |  |
| PTA (%) | 80-130 |  |
| APTT(s) | 25.1-36.5 |  |
| TT(s) | 10.3-16.6 |  |
| Fibrinogen(mg/dL) | 238-498 |  |
| D-dimer (ng/mL) | 0-500 |  |
| CRP (mg/L) | 0-10.0 |  |
| Procalcitonin (ng/mL) | 0-0.05 |  |
| ESR (mm/h) | 0-20 |  |
| SAA (mg/L) | 0-10 |  |
| Ferritin (ng/mL) | 5-204 |  |
| IL-2(pg/mL) | 0.1-4.1 |  |
| IL-4(pg/mL) | 0.1-3.2 |  |
| IL-6 (pg/mL) | 0.1-2.9 |  |
| IL-10 (pg/mL) | 0.1-5.0 |  |
| TNF-α (pg/mL) | 0.1-8.5 |  |
| IFN-γ (pg/ml) | 0.1-18.0 |  |
| CD3+ lymphocytes (%) | 50.0-84.0 |  |
| CD3+ lymphocytes (cells/ul) | 955-2860 |  |
| CD3+CD4+ lymphocytes (%) | 27.0-51.0 |  |
| CD3+CD4+ lymphocytes (cells/ul) | 550-1440 |  |
| CD3+CD8+ lymphocytes (%) | 15.0-44.0 |  |
| CD3+CD8+ lymphocytes (cells/ul) | 320-1250 |  |
| CD19+ lymphocytes (%) | 5.0-18.0 |  |
| CD19+lymphocytes (cells/ul) | 90-560 |  |
| CD16+CD56+ cells (%) | 7.0-40.0 |  |
| CD16+CD56+ cells (cells/ul) | 150-1100 |  |
