# Supplementary figures and images for: Age-related disparities in clinical characteristics and outcomes of patients with severe fever with thrombocytopenia syndrome
Source: PLoS Negl Trop Dis. 2025 Nov 4;19(11):e0013694. doi: 10.1371/journal.pntd.0013694 (PMC12604809; doi:10.1371/journal.pntd.0013694)

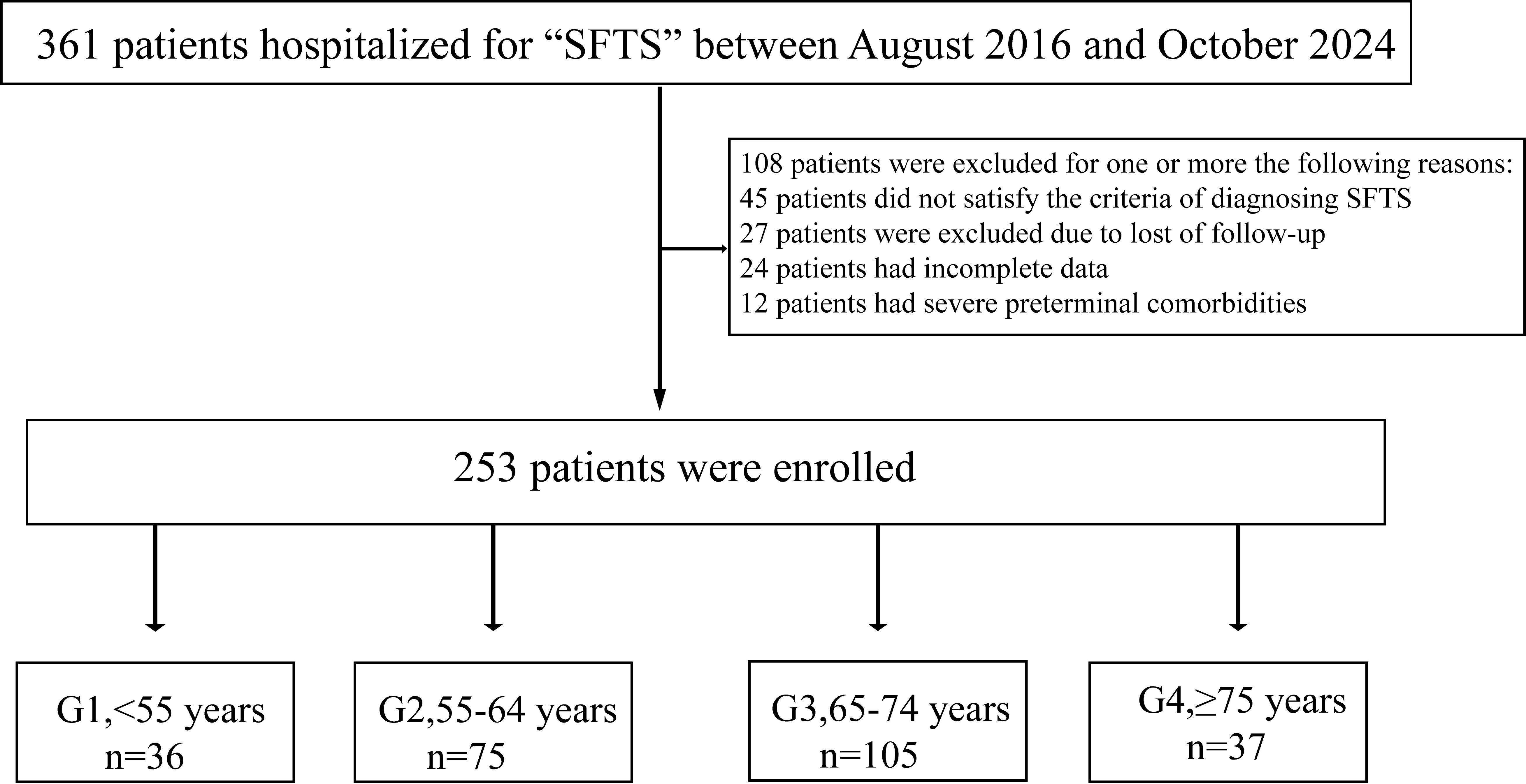

Supplement: S1 Fig — (TIF) [file pntd.0013694.s003.tif]
