# Supplementary material for: Age-related disparities in clinical characteristics and outcomes of patients with severe fever with thrombocytopenia syndrome
Source: PLoS Negl Trop Dis. 2025 Nov 4;19(11):e0013694. doi: 10.1371/journal.pntd.0013694 (PMC12604809; doi:10.1371/journal.pntd.0013694)
Supplement: Attachment — Submitted filename: Response to Reviewers.pdf [file pntd.0013694.s006.pdf]

General Comments:

Thank you for the opportunity to review your manuscript entitled "Age-related disparities in clinical characteristics and outcomes of patients with severe fever with thrombocytopenia syndrome." While the topic is important and clinically relevant, the manuscript has several major limitations in its design, analysis, and interpretation that preclude its acceptance in its current form.

Major Concerns:

Lack of Novelty and Contribution

The association between older age and poor outcomes in SFTS has already been established in previous studies. This manuscript largely confirms prior findings without providing novel insights or advancing the field.

Response:

We are grateful for your thoughtful critique regarding the perceived lack of novelty and contribution in our manuscript concerning the relationship between older age and poor outcomes in severe fever with thrombocytopenia syndrome (SFTS). We recognize that the association between age and SFTS outcomes has been previously established, and our study does, to some extent, corroborate these existing findings. Nevertheless, we would like to elucidate several aspects of our study that we believe contribute to the advancement of knowledge in this field.

First, we conducted a comprehensive analysis of the demographics, comorbid conditions, and clinical manifestations across different age groups of SFTS patients. Previous studies have often focused on the overall age-outcome relationship without a detailed breakdown of these factors. By examining how these characteristics vary with age, we have provided a more nuanced understanding of the disease profile in different age cohorts. For example, we found that older patients (age ≥65 years) had

higher serum levels of laboratory parameters referring to liver, kidney, cardiac, and coagulation system injury. It is implied that older patients with SFTS are more prone to multiple organ injury or even failure, which may contribute to their adverse outcomes. This information is crucial for clinicians to better assess the risk and tailor treatment strategies for patients of different ages.

Second, we delved into the immunological aspects of SFTS by analyzing immune cell counts and inflammation factor levels in different age groups. While some studies have touched upon the immune response in SFTS, our study is the first to specifically compare these parameters across age strata. We discovered that older patients exhibited distinct immune dysregulation patterns, with lower counts of certain immune cells and higher levels of inflammatory cytokines. These findings offer new insights into the age-related immune mechanisms underlying the poor prognosis in SFTS and may guide the development of immunomodulatory therapies.

In addition, we investigated the viral shedding time, platelet recovery time, and cumulative survival rate in different age groups. Previous research has not systematically compared these important clinical endpoints across age groups. Our study revealed that older patients (age ≥65 years) had longer viral shedding time, slower platelet recovery, and a significantly lower cumulative survival rate compared to younger patients (age <65 years). Notably, we found that age ≥65 years was identified as an independent risk factor for poor prognosis in SFTS. This is a critical finding that can help clinicians identify high-risk patients early and implement more aggressive interventions.

In conclusion, although our study confirms the existing age-outcome relationship in SFTS, it goes beyond previous work by providing a detailed analysis of demographics, comorbidities, clinical manifestations, immunological parameters, and key clinical

endpoints across different age groups. The identification of age ≥65 as an independent risk factor for poor prognosis is a significant contribution that can have important implications for clinical practice and public health interventions. We believe these novel findings make our study a valuable addition to the scientific literature on SFTS. Thank you again for your time and feedback.

Arbitrary Age Grouping Without Justification

The division into four age groups (≤54, 55–64, 65–74, ≥75) appears arbitrary. The rationale for these cutoffs is not explained, nor is there evidence that such stratification provides clinically meaningful distinctions.

Response:

Thank you very much for your insightful comments and constructive feedback on our manuscript. We truly appreciate the time and effort you have dedicated to reviewing our work, and we are grateful for the opportunity to address your concerns regarding the age-grouping in our study.

We acknowledge that the division into the four age groups (≤54, 55–64, 65–74, ≥75) may initially seem arbitrary without proper explanation. In fact, these cut-offs were not randomly chosen but were based on a combination of factors.

We conducted an extensive literature review before finalizing the age groups. Many previous studies in the relevant field have used similar age brackets to analyze age-related trends and differences. For example, in studies on age-related health conditions such as cardiovascular diseases (Lancet. 2021;398(10305):1053-1064; BMJ. 2021;372: n461), cancer (Eur J Cancer. 2004;40(15):2307-2316; Br J Cancer. 2005;93(3):372-375), diabetes (Diabetes Care. 2024;47(3):353-361; Lancet Healthy Longev. 2021;2(10): e618-e628), and cognitive decline (BMC Geriatr. 2019;19(1):59;

Ann Geriatr Med Res. 2024;28(1):65-75), these age ranges have been commonly employed to capture distinct stages of physiological and pathological changes. The age of 65 is often considered a significant milestone as it is a common retirement age in many countries and is associated with a shift in lifestyle, access to healthcare services, and the onset of age-related comorbidities. The 55-64 age group can be seen as a transitional period leading up to this milestone, while the 75 and above group represents a more advanced stage of aging with potentially more complex health profiles (*Adv Data*. 2006;(370):1-31).

We also took into account the distribution of our study population when determining the age groups. We aimed to create groups that had a sufficient number of participants to ensure statistical power and reliability of the results. If we had chosen narrower or different age intervals, some groups might have had too few cases, leading to unstable estimates and reduced generalizability of the findings. The current age grouping allowed us to balance the need for meaningful age-based comparisons while maintaining adequate sample sizes within each group.

To further support the clinical significance of our age-grouping, we have conducted additional post-hoc analyses. We examined the in-hospital mortality rates across the four age groups. The results showed significant differences between the groups, indicating that the age-based stratification can indeed capture important variations in outcomes. The in-hospital mortality rate increased steadily with age, from 5.6% in the <55 age group to 10.7% in the 55-64 age group, 25.7% in the 65-74 age group, and 27% in the ⩾75 age group ($p < 0.006$). The Kaplan-Meier survival curves showed clear separation between the age groups, indicating that there were significant differences in the probability of experiencing the primary outcome over time.

Once again, we thank you for your valuable feedback, which has helped us improve the quality and clarity of our manuscript. We hope that our response has adequately addressed your concerns.

Overwhelming Volume of Data Without Focus

The manuscript presents an overwhelming number of clinical and laboratory variables, many of which do not show significant differences or add value to the conclusions. This hinders readability and dilutes the main message. The analysis feels more descriptive than hypothesis-driven.

Response:

Thank you very much for taking the time to carefully review our manuscript and for providing such insightful feedback. We sincerely appreciate your constructive criticism, which will undoubtedly help us improve the quality and clarity of our work.

We understand your concern regarding the presentation of a large number of clinical and laboratory variables in the manuscript. We acknowledge that this may have made the analysis appear more descriptive than hypothesis-driven and could potentially hinder readability by diluting the main message. Our intention in including these variables was to provide a comprehensive overview of the study population and to ensure that no potentially relevant factors were overlooked.

First, we showed the difference in demographics, comorbid conditions, and clinical manifestations of patients across four age groups. Second, we demonstrated the disparities in laboratory test results and complications of patients across four age groups. We aimed to elucidate the reasons for the poor prognosis of SFTS patients aged ≥65 years from the differences in clinical and laboratory variables. In the results part, the variables we elaborated on in detail are those with significant differences

across the four age groups. In the discussion part, we also provided a detailed interpretation of these findings to explain why patients aged ≥65 years have a higher mortality rate. We are committed to ensuring that our work is presented in a clear and concise manner that effectively communicates the main findings and their implications. Once again, we thank you for your valuable feedback and for your role in improving the quality of our research.

Insufficient Control for Confounders

Although multivariate logistic regression was performed, it is unclear whether important confounders (such as comorbidities and baseline functional status) were adequately adjusted for. This weakens the strength of the conclusions, particularly regarding the role of age as an independent predictor.

Response:

We sincerely appreciate the time and effort you've dedicated to reviewing our manuscript and providing such insightful feedback. Your concern regarding the adequacy of adjusting for important confounders, specifically comorbidities and baseline functional status, in the multivariate logistic regression analysis is well-taken. We fully recognize that insufficient adjustment for these factors could indeed undermine the validity of our conclusions, especially concerning the role of age as an independent predictor. Below, we present a detailed response to address this issue.

In our initial analysis, we identified and included a set of common comorbidities based on a comprehensive literature review and clinical expertise in the field of infectious disease. These comorbidities encompassed major chronic conditions such as hypertension, diabetes, coronary heart disease, and chronic obstructive pulmonary disease (COPD). We collected data on these comorbidities from patients' medical

records, which included detailed admission notes, discharge summaries, and laboratory and imaging reports. Each comorbidity was treated as a binary variable in the univariate and multivariate logistic regression model.

For baseline functional status, we employed the Barthel Index (BI) to quantify patients' ability to perform daily living activities. This scale assesses multiple domains, including feeding, bathing, dressing, toileting, continence, and mobility. The total score ranges from 0 to 100, with higher scores indicating better functional status (Eur J Intern Med. 2018 Jan: 47:69-74). We established four categories: total dependency (BI ≤20), severe dependency (BI 21–40), moderate dependency (BI 41–60), and mild or no dependency (BI 61–100). Base functional status was also included in the univariate and multivariate logistic regression analyses.

The results confirmed that age ≥65 years remained the independent predictor of mortality even after accounting for comorbidities and baseline functional status, which were updated in Table 5. In conclusion, we deeply appreciate your valuable comment, which has prompted us to conduct a more thorough and rigorous analysis. By expanding the list of comorbidities and baseline functional status, we have significantly improved the quality of our study. The results of the improved analysis provide more reliable evidence regarding the role of age as an independent predictor, as well as the impacts of other factors on the outcome. We have updated the relevant sections of the manuscript, including the methods, results, and discussion, to reflect these changes and provide a more accurate and comprehensive presentation of our findings.

Limited Interpretation of Immune Findings

The reported differences in inflammatory cytokines and immune cell subsets are not adequately interpreted. The discussion lacks depth in exploring mechanisms such as immunosenescence and their relevance to viral pathogenesis and outcomes.

Response:

We are truly grateful for your detailed and constructive feedback on our manuscript. Your insights have been invaluable in helping us enhance the quality and depth of our work. We have carefully considered your comments and made the following revisions to address the issues you raised.

In the original version, we mainly presented the data on inflammatory cytokines (e.g., IL-6, IL-10, TNF-α) and immune cell subsets (CD3+ and CD3+CD4+ lymphocytes) without fully exploring their biological significance. For inflammatory cytokines, we now provide a more in-depth analysis. IL-6 is a key pro-inflammatory cytokine that plays a pivotal role in the acute-phase response. Elevated levels of IL-6 in our study can be interpreted as an indication of an active immune response against the virus. However, excessive IL-6 production may lead to a cytokine storm, which is associated with severe tissue damage and multi-organ failure, as has been observed in many viral infections (Lancet Respir Med. 2021 Jun;9(6):643-654). IL-10 is an anti-inflammatory cytokine, an increase in IL-10 could indicate an attempt by the body to control inflammation, but it may also impair the clearance of the virus (J Immunol Res. 2017:2017:6104054). TNF-α is a central mediator of inflammation and immune cell activation, and triggers an inflammatory response at the site of infection. Excessive production of TNF-α can contribute to the development of a cytokine storm, which can cause severe tissue damage, leading to multi-organ failure. (Nat Rev Immunol. 2023 May;23(5):289-303).

Regarding immune cell subsets, CD3+ lymphocytes are a broad category of T cells, which are crucial for cell-mediated immunity. CD3+CD4+ lymphocytes, also known as helper T cells, play a central role in coordinating the immune response by secreting cytokines that activate other immune cells. A decrease in CD3+ and CD3+ CD4+ lymphocytes may indicate an impaired immune response, making the patients more susceptible to severe viral infection and less able to clear the virus (Signal Transduct Target Ther. 2023 Jun 19;8(1):235).

In the context of our study, immunosenescence can help explain the differences in viral pathogenesis and outcomes among different age groups. Elderly patients, who are more likely to experience immunosenescence, may have a less effective immune response against the virus, leading to a higher viral load, more severe symptoms, and a greater risk of complications. This is consistent with our observation that elderly patients (age ≥65 years) in our cohort had adverse clinical outcomes compared to younger patients (age <65 years).

We have incorporated these detailed interpretations and discussions into the revised manuscript. We hope that these changes will address your concerns and make the manuscript more suitable for publication. Thank you again for your time and valuable feedback.

Redundancy With Authors' Prior Publications

Many elements of the data and discussion appear to overlap with the authors' previous work (e.g., studies on DIC, AKI, SIRS in SFTS). The manuscript could be perceived as a repackaging of prior analyses with a new framing, which raises concerns about publication novelty.

Response:

Thank you for taking the time to carefully review our manuscript and for providing these insightful comments. We sincerely appreciate your concern regarding the potential perception of our work as a mere repackaging of prior analyses with a new framing, and we would like to address this issue comprehensively.

We acknowledge that there are apparent overlaps between some elements of the data and discussion in this manuscript and our previous studies on DIC, AKI, and SIRS in SFTS. However, we would like to emphasize that this current work represents a significant advancement and is not simply a rehash of past research. We showed the difference in these complications, including DIC, AKI, and SIRS, among patients across four age groups. It was confirmed that patients aged ≥65 years had a higher proportion of DIC, AKI, and SIRS than patients aged <65 years. These complications were proven to be associated with high mortality of patients with SFTS. In our study, we aimed to provide a reasonable theoretical basis for explaining the poor prognosis of older patients (age ≥65 years) based on our previous studies.

In conclusion, while there may be some overlaps with our previous research, we firmly believe that this manuscript presents a substantial and original contribution to the field of SFTS research. The new research questions, enhanced analytical methods, and broader clinical implications distinguish this work from our prior studies and underscore its novelty and significance. We hope that these explanations and revisions have adequately addressed your concerns. Thank you again for your valuable time and constructive comments.

Minor Comments:

The writing requires substantial editing for grammar, clarity, and flow.

Abbreviations such as DIC, SIRS, HLH should be defined at first mention.

Figures (especially Kaplan–Meier curves) require improved labeling and resolution.

The abstract is overly long and should be more concise.

Response: We sincerely thank you for pointing out this crucial aspect. We have conducted a comprehensive review of the entire manuscript and made the following improvements.

We have meticulously checked each sentence for grammatical errors, including subject-verb agreement, tense consistency, and proper use of articles and prepositions. All identified errors have been corrected to ensure grammatical accuracy throughout the text. We also have rephrased complex and ambiguous sentences to make the meaning more explicit. For example, in sections where technical concepts were presented in a convoluted manner, we have broken them down into simpler, more understandable statements. Additionally, we have restructured several paragraphs and sections to improve the overall flow of the manuscript. We have also ensured that each paragraph has a clear topic sentence and supporting details, which helps in maintaining a consistent and focused discussion.

We acknowledge the importance of defining abbreviations for the benefit of all readers, especially those who may not be familiar with the specific medical or scientific terms. We have now included the full forms of DIC (disseminated intravascular coagulation), SIRS (systemic inflammatory response syndrome), and HLH (hemophagocytic lymphohistiocytosis) at their first appearance in the text (page 6, lines 123-125). This ensures that readers can immediately understand the meaning of these abbreviations without having to search for definitions elsewhere, thereby improving the overall readability and accessibility of the manuscript.

We have revised the labels on all figures, with particular emphasis on the Kaplan-Meier curves. Each axis is now clearly labeled with the appropriate variable name and

unit of measurement. We have increased the resolution of all figures, including the Kaplan-Meier curves, to ensure that they are sharp and clear, even when printed or viewed at high magnification. We have also optimized the file formats to prevent any loss of quality during the submission or publication process.

We have carefully reviewed each sentence in the abstract and eliminated any repetitive or non-essential details. We have condensed the results section to highlight only the most important and impactful findings. Instead of presenting detailed numerical data, we have provided a general overview of the trends and significant outcomes. We have rephrased the conclusion to make it more focused and impactful, emphasizing the main implications and contributions of our study.
