# Supplementary material for: Age-related disparities in clinical characteristics and outcomes of patients with severe fever with thrombocytopenia syndrome
Source: PLoS Negl Trop Dis. 2025 Nov 4;19(11):e0013694. doi: 10.1371/journal.pntd.0013694 (PMC12604809; doi:10.1371/journal.pntd.0013694)
Supplement: Attachment — Submitted filename: Response_to_Reviewers_auresp_2.pdf [file pntd.0013694.s007.pdf]

Reviewer #2: IL-8 and IFN-α levels also were higher and TGF-β levels were lower in patients with fatal disease than in patients with nonfatal disease during the initial clinical course of SFTS (Ref. Fatal outcome of severe fever with thrombocytopenia syndrome (SFTS) and severe and critical COVID-19 is associated with the hyperproduction of IL-10 and IL-6 and the low production of TGF-β. J Med Virol. 2023 Jul;95(7): e28894). Could authors also show data of these cytokines (IL-8, IFN-α, and TGF-β) in this study?

Response:

Thank you for your insightful comment regarding the cytokine levels (IL-8, IFN-α, and TGF-β) in our study. We acknowledge the importance of these cytokines in understanding the disease progression and outcomes, as highlighted by the reference to the study published in the Journal of Medical Virology.

However, we would like to clarify that our study is a retrospective analysis, and as such, the serum levels of IL-8, IFN-α, and TGF-β were not routinely measured in our patient cohort. Due to this limitation, we are unable to present the relevant data on these cytokines in our current manuscript.

We recognize the potential value of incorporating these cytokine measurements in future prospective studies to further elucidate the immunological mechanisms underlying the disease severity and outcomes. We appreciate your suggestion and will consider it for our future research endeavors.

Thank you again for your constructive feedback, which helps us improve the quality and depth of our research.

Reviewer #4: The authors didn't response my comments.

Response:

We sincerely apologize for the previous oversight in not responding to your comments. We greatly value your input and have carefully considered each of your points. Below, we provide detailed responses to your comments:

1.Does the patient only need to meet one of the criteria or meet all three criteria simultaneously?

We would like to clarify that in this present study, patients were required to meet all three specified criteria for inclusion. This was to ensure a homogeneous study population and to enhance the reliability of our findings. The criteria were designed to capture patients with specific characteristics relevant to our research objectives.

2. Please clarify the exact time point of specimen collection relative to admission in the study protocol.

Thank you for pointing out the need for clarification. In our study, specimens were collected within 24 hours of patient admission. This time frame was chosen to minimize the variability in specimen quality and to ensure that the samples reflected the patient's condition at the time of presentation. We have now updated the study protocol section in the manuscript to explicitly state this time point.

3. The table 5 lacks proper citations in the main text

We apologize for this oversight. Table 5 has been revised to include proper citations in the main text. We have cross-referenced each data point in the table with the corresponding section in the manuscript to ensure clarity and traceability.

4. SFTSV has been renamed Dabie bandavirus

We appreciate your update on the nomenclature. In the revised manuscript, we updated it in the Introduction part (page 3, line 50).

5. the transmission of person to person didn't cause the increase of patients

We agree with your kind comments, and the description "As SFTSV can be transmitted from person to person, the number of SFTSV infection cases has steadily increased in recent years, making it a globally emerging infectious disease" was corrected into "The increasing incidence of SFTS has been linked to the expanding range of virus-carrying tick vectors and a growing number of human-to-human transmission via multiple routes, making it a globally emerging infectious disease".

Reviewer #5: The authors described the differences in the clinical characteristics, including demographics, comorbidities, clinical symptoms, laboratory test results, complications, and treatment in severe fever with thrombocytopenia syndrome (SFTS) patients across four age groups. Certain citations in the Introduction section require greater precision, and the Discussion section would benefit from a more in-depth analysis of potential mechanisms.

Response:

We sincerely appreciate your detailed and constructive feedback on our manuscript. We have carefully considered each of your comments and have made the necessary revisions to

improve the quality and clarity of our work. Below, we provide point-by-point responses to your comments:

1. Line 52-55: The increasing incidence of SFTS has been linked to the expanding range of virus-carrying tick vectors and a growing number of human-to-human transmission via multiple routes. Ref: Clin Infect Dis 2021 PMID: 33068430, Int J Infect Dis 2022, PMID: 35987469, etc.
Thank you for highlighting the need for more precise citations. According to your kind comments, we have updated the description and references in our manuscript (page 3, line 52-55).

2.Line 60: I recommend citing studies that include larger patient cohorts or multi-center data sources, but not from the same center.
We agree with your recommendation. We have revised the citations in this section to include studies with larger patient cohorts and multi-center data sources, ensuring a broader representation of the SFTS patient population.

3.Line 61: Please identify studies that either support or refute the association between age and SFTS incidence.

The original statement ("age is not a risk factor for SFTSV infection") refers to **acquisition of infection** (i.e., exposure to SFTSV-carrying ticks), which may not differ significantly by age. However, as you correctly noted, **SFTS disease onset and severity** are strongly age-dependent. This distinction is critical and has been emphasized in the revised manuscript (page 3, line 62-63).

4.Line 81: Is there a specific rationale or particular consideration behind the authors' use of this age-stratification approach?

Thank you very much for your insightful comments and constructive feedback on our manuscript. We truly appreciate the time and effort you have dedicated to reviewing our work, and we are grateful for the opportunity to address your concerns regarding the age-grouping in our study.

We acknowledge that the division into the four age groups (≤54, 55–64, 65–74, ≥75) may initially seem arbitrary without proper explanation. In fact, these cut-offs were not randomly chosen but were based on a combination of factors.

We conducted an extensive literature review before finalizing the age groups. Many previous

studies in the relevant field have used similar age brackets to analyze age-related trends and differences. For example, in studies on age-related health conditions such as cardiovascular diseases (Lancet. 2021;398(10305):1053-1064; BMJ. 2021;372: n461), cancer (Eur J Cancer. 2004;40(15):2307-2316; Br J Cancer. 2005;93(3):372-375), diabetes (Diabetes Care. 2024;47(3):353-361; Lancet Healthy Longev. 2021;2(10): e618-e628), and cognitive decline (BMC Geriatr. 2019;19(1):59; Ann Geriatr Med Res. 2024;28(1):65-75), these age ranges have been commonly employed to capture distinct stages of physiological and pathological changes. The age of 65 is often considered a significant milestone as it is a common retirement age in many countries and is associated with a shift in lifestyle, access to healthcare services, and the onset of age-related comorbidities. The 55-64 age group can be seen as a transitional period leading up to this milestone, while the 75 and above group represents a more advanced stage of aging with potentially more complex health profiles (Adv Data. 2006;(370):1-31).

We also took into account the distribution of our study population when determining the age groups. We aimed to create groups that had a sufficient number of participants to ensure statistical power and reliability of the results. If we had chosen narrower or different age intervals, some groups might have had too few cases, leading to unstable estimates and reduced generalizability of the findings. The current age grouping allowed us to balance the need for meaningful age-based comparisons while maintaining adequate sample sizes within each group.

To further support the clinical significance of our age-grouping, we have conducted additional post-hoc analyses. We examined the in-hospital mortality rates across the four age groups. The results showed significant differences between the groups, indicating that the age-based stratification can indeed capture important variations in outcomes. The in-hospital mortality rate increased steadily with age, from 5.6% in the <55 age group to 10.7% in the 55-64 age group, 25.7% in the 65-74 age group, and 27.0% in the ⩾75 age group ($p < 0.006$). The Kaplan-Meier survival curves showed clear separation between the age groups, indicating that there were significant differences in the probability of experiencing the primary outcome over time.

Once again, we thank you for your valuable feedback, which has helped us improve the quality and clarity of our manuscript. We hope that our response has adequately addressed your concerns.

5. Line 122-125: The diagnostic criteria for SIRS, DIC, and HLH.

We have clarified the diagnostic criteria for SIRS (Systemic Inflammatory Response Syndrome), DIC (Disseminated Intravascular Coagulation), and HLH (Hemophagocytic

Lymphohistiocytosis) in this section. We have provided specific criteria and references to ensure clarity and accuracy (page 6-7, line 128-135).

6. Line 131-133: Detailed information regarding the follow-up, including the methodology and time frame, is requested.

We have expanded the description of the follow-up methodology and time frame in this section. We now provide detailed information on how patients were followed up, including the frequency of visits, the duration of follow-up, and the endpoints assessed (page7, line 140-146).

7. Figures 2 and 3: The number of patients should be indicated at each time point. Why are the follow-up times different?

We sincerely appreciate the reviewer's insightful suggestion. We fully agree that indicating the number of patients at each time point in Figures 2 and 3 would significantly enhance the clarity and comprehensibility of our data presentation. In the revised manuscript, we have carefully added the specific number of patients corresponding to each time point in both figures. This additional information allows readers to better understand the sample size dynamics over the course of the study, facilitating a more accurate interpretation of the results. Figure 2A and 2B showed the SFTSV RNA positivity rate and platelet count normalization rate of patients at different follow-up days after hospitalization, respectively. Figure 3 demonstrated the cumulative survival rates of patients at different follow-up days after hospitalization. These two figures displayed three parameters at different follow-up times, so the follow-up time points were different.

8. The conclusion are supported by the data presented. Other limitations should be described, such as single center based analysis.

We agree with your comments. We have now included a discussion of the limitations of our study, including the fact that it was based on a single center. We acknowledge that this may limit the generalizability of our findings and suggest that multi-center studies are needed to confirm our results (page 21, line 353-355).

9. Discussion: The authors should discuss the potential mechanisms behind the differences in susceptibility to SFTSV and in antiviral immune responses across age groups, which may also

apply to other similar hemorrhagic fever viruses. Additionally, it is important to note the higher proportion of males in the two older age groups, especially since male sex is associated with a poorer prognosis following SFTSV infection. The relatively small sample size may also lead to confounding effects related to gender.

We have expanded the Discussion section to include a more in-depth analysis of the potential mechanisms behind age-related differences in susceptibility to SFTSV and antiviral immune responses. We discuss how these mechanisms may also apply to other hemorrhagic fever viruses (page 19-20, line 314-334). Additionally, we have noted the higher proportion of males in the older age groups, which agreed with other reports (J Med Virol. 2023 Mar;95(3): e28632; Front Microbiol. 2021 Oct 14:12:738808). In our study, the in-hospital mortality rates of males and females were 17.9% and 19.2%, respectively. There is no statistical difference in in-hospital mortality rate between males and females. Therefore, we only reveled male predominance in older patients (age ≥65 years), but could not prove that male sex is associated with a poorer prognosis following SFTSV infection. According to your kind comments, these details were updated in the Discussion section (page 18, line 278-283).

Reviewer #6: The author compared the immune status of different age groups, which was kind of novel. But the analysis of the population feels more descriptive than hypothesis-driven. The strength of the conclusions were weak without providing novel insights. The design of the manuscript did not match the conclusion very well.

Response:

We sincerely appreciate the time and effort you've dedicated to reviewing our manuscript and for providing such insightful and constructive comments. You correctly pointed out that our initial analysis of the population leaned more towards descriptive statistics. In the early stages of the study, our primary objective was to comprehensively map out the immune status across different age groups, aiming to establish a foundational understanding of the general immune landscape in this context. However, we fully acknowledge that a descriptive approach alone is insufficient for advancing scientific knowledge in a meaningful way. According to your kind comments, we stated that SFTS patients aged 65 and above were more prone to impaired immune function in the Conclusion part. We also emphasized that immune function was weakened with age in the context of SFTS (page 19-20, line 315-335).
