# Supplementary material for: Age-related disparities in clinical characteristics and outcomes of patients with severe fever with thrombocytopenia syndrome
Source: PLoS Negl Trop Dis. 2025 Nov 4;19(11):e0013694. doi: 10.1371/journal.pntd.0013694 (PMC12604809; doi:10.1371/journal.pntd.0013694)
Supplement: Attachment — Submitted filename: Response_to_Reviewers_auresp_3.pdf [file pntd.0013694.s008.pdf]

Reviewer #5: The authors should acknowledge the subjectivity of age stratification and its potential impact on the analysis in the discussion section.

Response:

We appreciate your insightful comment regarding the subjectivity of age stratification and its potential impact on our analysis. In response to this comment, we have incorporated a discussion on the subjectivity of the age stratification used in our study and its potential implications in the Discussion section, as follows:

"We also recognize the subjectivity inherent in the age stratification employed in our study, where patients were categorized into four distinct groups based on age thresholds. This stratification was primarily guided by the distribution of ages among the included SFTS patients and aimed to facilitate a meaningful comparison of clinical characteristics and outcomes across different life stages. However, we recognize that such stratification is somewhat arbitrary and may not fully capture the continuous and nuanced relationship between age and disease severity. The impact of age on SFTS severity may not be linear, and other unmeasured factors could interact with age in complex ways, affecting the prognosis. Future studies could employ more sophisticated statistical methods to explore the continuous relationship between age and SFTS outcomes. Additionally, considering other relevant demographic and clinical variables in multivariate models can help disentangle the independent effects of age on disease severity."

Despite these limitations, our current stratification approach provides valuable insights into the age-related disparities in SFTS patients and highlights the need for tailored management strategies for different age groups. We hope this revised discussion adequately addresses your concerns and enhances the overall robustness of our manuscript.

Reviewer #6: I think this article provides a detailed description of the immune indicators of SFTS patients, which offers certain basis for explaining the clinical symptoms of these patients. This is very good.

Response:

Thank you for your thoughtful comments on our manuscript, and we greatly appreciate your recognition of the detailed description of immune indicators in patients with SFTS.

Indeed, our goal was to provide a comprehensive analysis of how immune system alterations in SFTS patients correlate with their clinical symptoms, and we are pleased that you find this aspect valuable. In our study, we observed significant age-related differences in immune function among SFTS patients. Specifically, older patients (aged ≥65 years) exhibited markedly lower percentages and counts of CD3+ and CD3+CD4+ lymphocytes compared to younger patients (aged <65 years). These T cell subsets play a pivotal role in orchestrating immune responses against viral infections. The reduction in these lymphocytes suggests an impaired immune response in elderly patients, which may contribute to their increased susceptibility to severe viral infections and difficulty in clearing the virus effectively.

Moreover, our data revealed that older patients had significantly higher serum levels of procalcitonin, serum amyloid A (SAA), ferritin, interleukin-6 (IL-6), interleukin-10 (IL-10), and tumor necrosis factor-α (TNF-α). These acute-phase proteins and inflammatory cytokines are critical in the body's response to infection but can also lead to tissue damage and multi-organ failure when produced in excess. For instance, IL-6, a key pro-inflammatory cytokine, can drive a cytokine storm, causing severe tissue damage, while IL-10, an anti-inflammatory cytokine, may impair viral clearance. TNF-α, another central mediator of inflammation, can contribute to the development of a cytokine storm and subsequent multi-organ failure.

These immune alterations provide a mechanistic basis for explaining why older SFTS patients are more prone to multiple organ dysfunction and have a higher risk of

adverse outcomes. The elevated inflammatory response, coupled with an impaired immune response, likely contributes to the severity of clinical symptoms observed in these patients, such as lethargy, chest distress, dyspnea, gingival bleeding, and myasthenia.

We believe that by elucidating these immune indicators and their clinical implications, our study offers important insights for clinicians managing SFTS patients. Tailored treatment strategies that consider age-related immune differences may be necessary to improve patient outcomes. Once again, we thank you for your positive feedback and constructive comments.
