# Supplementary material for: Age-related disparities in clinical characteristics and outcomes of patients with severe fever with thrombocytopenia syndrome
Source: PLoS Negl Trop Dis. 2025 Nov 4;19(11):e0013694. doi: 10.1371/journal.pntd.0013694 (PMC12604809; doi:10.1371/journal.pntd.0013694)
Supplement: Attachment — Submitted filename: Reviewers Comment.docx [file pntd.0013694.s004.docx]

**General Comments:**

Thank you for the opportunity to review your manuscript entitled "Age-related disparities in clinical characteristics and outcomes of patients with severe fever with thrombocytopenia syndrome." While the topic is important and clinically relevant, the manuscript has several major limitations in its design, analysis, and interpretation that preclude its acceptance in its current form.

**Major Concerns:**

**Lack of Novelty and Contribution**

The association between older age and poor outcomes in SFTS has already been established in previous studies. This manuscript largely confirms prior findings without providing novel insights or advancing the field.

**Arbitrary Age Grouping Without Justification**

The division into four age groups (≤54, 55–64, 65–74, ≥75) appears arbitrary. The rationale for these cutoffs is not explained, nor is there evidence that such stratification provides clinically meaningful distinctions.

**Overwhelming Volume of Data Without Focus**

The manuscript presents an overwhelming number of clinical and laboratory variables, many of which do not show significant differences or add value to the conclusions. This hinders readability and dilutes the main message. The analysis feels more descriptive than hypothesis-driven.

**Insufficient Control for Confounders**

Although multivariate logistic regression was performed, it is unclear whether important confounders (such as comorbidities and baseline functional status) were adequately adjusted for. This weakens the strength of the conclusions, particularly regarding the role of age as an independent predictor.

**Limited Interpretation of Immune Findings**

The reported differences in inflammatory cytokines and immune cell subsets are not adequately interpreted. The discussion lacks depth in exploring mechanisms such as immunosenescence and their relevance to viral pathogenesis and outcomes.

**Redundancy With Authors’ Prior Publications**

Many elements of the data and discussion appear to overlap with the authors’ previous work (e.g., studies on DIC, AKI, SIRS in SFTS). The manuscript could be perceived as a repackaging of prior analyses with a new framing, which raises concerns about publication novelty.

**Minor Comments:**

The writing requires substantial editing for grammar, clarity, and flow.

Abbreviations such as DIC, SIRS, HLH should be defined at first mention.

Figures (especially Kaplan–Meier curves) require improved labeling and resolution.

The abstract is overly long and should be more concise.
